# Correction: Masking thiol reactivity with thioamide, thiourea, and thiocarbamate-based MBPs

**DOI:** 10.1039/d3cc90087h

**Published:** 2023-03-13

**Authors:** Hyeonglim Seo, Alysia J. Kohlbrand, Ryjul W. Stokes, Jeewon Chung, Seth M. Cohen

**Affiliations:** a Department of Chemistry and Biochemistry, University of California, San Diego 9500 Gilman Drive La Jolla CA 92093 USA scohen@ucsd.edu

## Abstract

Correction for ‘Masking thiol reactivity with thioamide, thiourea, and thiocarbamate-based MBPs’ by Hyeonglim Seo *et al.*, *Chem. Commun.*, 2023, **59**, 2283–2286, https://doi.org/10.1039/D2CC06596G.

The authors regret that in Fig. 1 of the original article the methyl-group of l-cysteine methyl ester (**13**) is incorrectly drawn. The corrected figure is shown here.

**Fig. 1:**
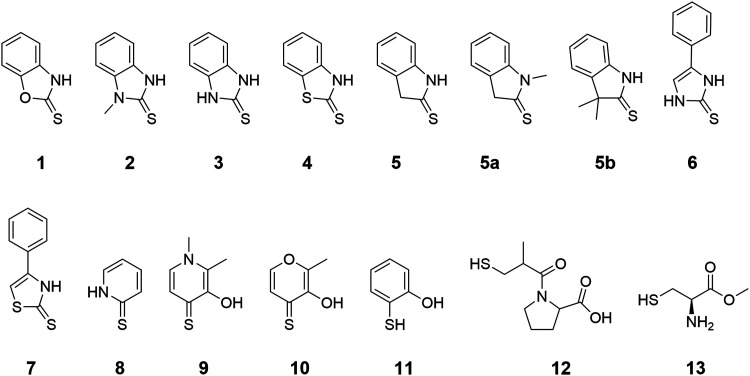
Thioamide, thiourea, and thiocarbamate MBPs proposed for use in Zn(ii)-dependent metalloenzymes. Compounds **9** and **10** were utilized as known thione-based MBPs and 2-mercaptophenol (**11**), captopril (**12**), and l-cysteine methyl ester (**13**) were used as representative thiol-based compounds in this paper.

The Royal Society of Chemistry apologises for these errors and any consequent inconvenience to authors and readers.

## Supplementary Material

